# Non-Nutritive (Artificial) Sweetener Knowledge among University Students

**DOI:** 10.3390/nu11092201

**Published:** 2019-09-12

**Authors:** Ted Wilson, Breanna Murray, Tamara Price, Denzel Atherton, Tisha Hooks

**Affiliations:** 1 Department of Biology, Winona State University, Winona, MN 55987, USA; brmmurray15@winona.edu (B.M.); tprice14@winona.edu (T.P.); datherton15@winona.edu (D.A.); 2 Department of Mathematics and Statistics, Winona State University, Winona, MN 55987, USA; thooks@winona.edu

**Keywords:** non-nutritive sweetener, low calorie sweetener, artificial sweetener, aspartame, acesulfame, saccharin, sucralose, rebaudioside, mogroside, stevia

## Abstract

This study determined non-nutritive sweetener (NNS; artificial sweetener) depth of knowledge among university health and science students. An online survey was delivered to 1248 science students and completed by 493 respondents (19.0 ± 2.2 years old), evaluating ability to provide an NNS description/definition, examples of NNS from memory, and evaluate NNS word familiarity with a click-drag-box to identify six NNS by chemical name (CN) and six NNS by trade name (TN), relative to six decoy NNS, six caloric sweeteners, and six food items (mean ± standard deviation). NNS definitions contained 1.1 ± 1.1 of four previously defined elements suggestive of knowledge depth, with highest scores among self-described non-NNS users and food ingredient label users. Knowledge depth was not correlated with gender, age, American College Test score, or history of weight loss attempts. Without prompting, respondents could name 0.9 ± 1.1 NNS from memory, with highest scores among self-described non-NNS users (1.4 ± 0.8) and food ingredient label users (1.4 ± 0.8). NNS example memory was not correlated with gender, age, ACT score, or history of weight loss attempts. With the click-drag-box exercise, NNS were correctly identified 4.9 ± 1.0 times by TN and significantly less by CN (3.9 ± 1.9 times). Decoy NNS were incorrectly identified as being a real NNS 4.7 ± 1.3 times, while caloric sweeteners and food items were incorrectly identified as NNS 1.7 ± 1.7 times and 1.0 ± 1.5 times, (TN and Decoy NNS > CN > caloric sweetener and food item). NNS knowledge among university students may be inadequate for understanding what NNS are, if they consume NNS, or whether NNS are important for dietary health.

## 1. Introduction

Non-nutritive sweeteners (NNS; artificial sweeteners) have few or no calories, and little or no nutritional value. Organizations providing NNS use recommendations for reducing dietary carbohydrate intake include the Academy of Nutrition and Dietetics [1], American Diabetes Association [2], and the American Heart Association [2]. NNS are used extensively in countless beverages, food products ranging from popsicles to cookies, and non-food products ranging from toothpaste to electronic cigarettes.

Specific NNS can be identified by their chemical name (CN) or trade name (TN). For the purposes of this study, TN refers to the trademark or common name of the NNS-containing raw plant used as an ingredient. While CN describes the structure of the specific chemical or molecule responsible for the sweet taste. The TN is generally used for marketing purposes and appears on the package label, while the CN typically appears on the ingredient label, although the name of a raw plant may also appear on an ingredient label if the plant is added in raw form (i.e., stevia leaf or monk fruit). The CNs of several common NNS in the US market include acesulfame, aspartame, mogroside, rebaudioside, saccharin and sucralose. Their respective TN’s are SweetOne^®^, Equal^®^, Monk fruit, Stevia, Sweet’N Low^®^, and Splenda^®^.

While NNS ingredients on food and beverage products can facilitate “low sugar” or “low calorie” packaging claims that may increase customer-product appeal, these packaging claims may not be associated with improved dietary health or nutritional knowledge [3]. NNS use adherent to US Food and Drug Administration (FDA) guidelines is generally assumed to be safe for human consumption, although their assumed utility for weight management, cardiovascular disease, and mortality has been questioned by some investigators [4,5,6]. However, the question of relative NNS safety or efficacy for weight control is not the focus of this study and will need to be further addressed by other investigators.

While the monetary value of the NNS industry is difficult to determine, it has been suggested that the market value will reach $13.4 billion globally by 2023 [7]. Changes in the use of NNS in the diet have been estimated by matching product purchase universal package code (UPC) scan data to produce ingredient lists [8,9]. Furthermore, analysis of two-day dietary recall in the National Health and Nutrition Examination Survey (NHANES 2009–2012) suggests that relative to 1999–2000 consumption of NNS among children and adults increased 200% and 54%, respectively [10]. Growth suggests changes in consumer preferences towards inclusion of more NNS containing beverages and foods. Nutrient labeling is intended to help consumers make informed decisions about their diet, but this may be counterproductive if the information is not correctly interpreted [11]. Several food label changes have been made by the FDA [12], so it is important to understand how well current food label information is understood by consumers to interpret the success of these changes. 

The term “diet soda” can imply that consumption is associated with dieting. The Fall 2018 American College Health Association survey showed that 36.0% of the 19,664 respondents indicated they were trying to lose weight [13]. A history of attempted prior weight maintenance may be associated with the use of reduced calorie foods and beverages, which should be suggestive of NNS knowledge [14]. However, in a survey of why people consume NNS, 31% of respondents suggested that they did not know enough about NNS to complete the survey [15]. Understanding consumer ability to identify NNS is important for describing how this population uses package labeling and ingredient lists. This consideration is important for understanding changes in NNS use [16] and whether NNS use is associated with improved health.

Given the popularity of NNS, there is a surprising paucity of data examining NNS understanding among university students. Knowledge of NNS is critical for the consumer to understand whether they consume NNS and which NNS they are consuming, presuming the consumer takes the time to read the ingredients list on a food label or examine food packaging information. The aim of this study of university health and science students was to use an online survey to determine their NNS depth of knowledge and ability to identify NNS examples. 

## 2. Materials and Methods

### 2.1. Participants and Study Design

The Winona State University Human Subjects Committee fully reviewed and approved this study prior to initiation (#1097547-1). All subjects gave their informed consent before participating in the study. All subjects (*n* = 1248) were enrolled in at least one of following freshman-level courses: Anatomy and Physiology (Bio 211 and Bio 212), Principles in Biology (Bio 241 and Bio 242), Personal and Community Health (HERS 204), General, Organic and Biochemistry (Chem 210), and Principles of Chemistry I and II (Chem 212 and Chem 213). Students were given a standardized 60-s presentation at the beginning of their lecture class (11 September 2017) regarding a “nutrition” survey that they were asked to voluntarily complete online. The presentation did not provide participants with survey content details, references to NNS, or other information that could create bias within the outcomes of the study. Lecture instructors were specifically asked to not discuss the survey in their lecture or to answer/address any questions about the survey or discuss NNS until the survey was completed. Participants in these classes were sent a single email (Monday 15:00; 11 September 2017) with a link to an online Qualtrics survey (Qualtrics LLC, Provo, UT, USA) and guaranteed complete anonymity with regards to their results. Investigators sent a single email reminder the next day (15:00), and the survey was closed on Wednesday 13 September 2017 at 15:00. The ballot-stuffing tool was used in Qualtrics to prevent participants from repeating the same survey more than once, and respondents were unable to go back and change answers in this sequential survey. 

### 2.2. Survey Administration, Content, and Flow

**Figure 1:**
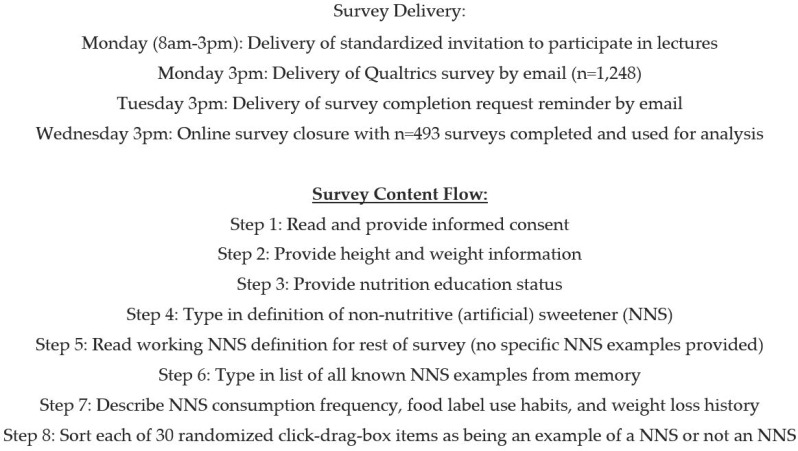
Sequence of online survey delivery and content flow.

The survey flow is outlined in Figure 1. Respondents opening the email link to this Qualtrics survey were first asked to provide information about their height, weight, and formal nutrition education with American College Test (ACT) score being obtained from student records in the university computer database (WSU-IPARS). Respondents were then asked to type a written definition of the term “non-nutritive (artificial) sweetener” into a textbox provided to evaluate respondent depth of knowledge regarding NNS. The investigators used four common elements used to describe/define NNS in prior investigations to create a working NNS definition for the present investigation [1,2,4,7]: *Non-Nutritive (artificial) Sweeteners are ingredients or chemicals that give a sweet taste to foods and beverages*, *have no nutritive benefits such as those from vitamins and protein*, *and have few or no calories*. This working definition sought to include the following qualities: (1) chemical nature, (2) taste, (3) caloric content, and (4) non-nutritive quality.

Respondent NNS definitions were graded by four study investigators, three of whom had to agree on whether each item met one of the four NNS qualities. Written definitions were graded on a scale of 0–4 points, with a score of 4 being a definition that correctly identified each of the four definition qualities and a score of 0 indicating that the definition contained none of the qualities or a response of “I don’t know”. Respondents earning four NNS definition quality points presumably had a rich knowledge depth of NNS. Respondents with a score of “0” presumably had little knowledge depth of NNS or little confidence in their ability to provide an accurate NNS definition. 

After providing their NNS definition from memory (no prompting), respondents were asked to read the survey’s working NNS definition for clarification in order to properly complete the remainder of the survey. Respondents were asked to type into a Qualtrics input box (from memory) the names of all the NNS that they could remember. The same four study investigators examined NNS examples from respondents; if the word provided by the respondent was spelled identically or phonetically similar to a known NNS (TN or CN), it was considered acceptable. Words written that were not correct or phonetically similar to a known NNS were not considered to be a NNS. Agreement between three of the same four study investigators was required for each potential NNS identification. Respondents were then asked to complete questions about their history of NNS use, ingredient list/food label use, and weight loss habits. These were identified as three potentially confounding variables that could be predictive of respondent NNS depth of knowledge and definition depth score.

The last survey activity required completion of a click-drag-box exercise that included six NNS identified by TN, six respective NNS identified by CN, six words intended to “sound” like a possible NNS (decoy NNS words), six caloric sweeteners, and six common food items. An extensive list of decoy NNS names was created by the investigators to help understand how a successful product name could influence perceived NNS recognition by a respondent and how this could influence apparent NNS knowledge. Of the 30 decoy names created by the investigators, each potential decoy NNS name was evaluated using online search engines (Google.com, Yahoo.com, Google Scholar, and PubMed) for the usage of each word on the internet (online decoy NNS searches completed 2 May 2017). Decoy names found to be identical to, associated with, or phonetically similar to an existing beverage/food product, a copyright name, or found to be trademarked were not used in this survey. Potential decoy NNS names that were phonetically similar to any true NNS were also removed from this study. The six decoy names used in the present survey were: Aquasweetener, Glucataste, Murina, Neotaste-S, Rubralose, and Xana Light. Six caloric sweeteners were included to evaluate basic nutrition understanding: dextrose, fructose, glucose, starch, sucrose, and sugar. Six additional words that were suggestive of a raw food item, but words that most people would not consider a NNS (bacon, chicken, lard, mustard, protein, and table salt), were also included in the survey. The order of these survey words within the click-drag-box was randomly populated in each Qualtrics survey delivered to potential respondents in order to prevent possible bias due to word order placement.

### 2.3. Statistical Analysis

Statistical analysis was performed using JMP Pro 13 (SAS Institute Inc., Cary, NC, USA, 2016). Numerical variables were described with the mean ± standard deviation (SD). Categorical variables were described in terms of their relative frequency. A one-way ANOVA was used to compare definition scores across groups, and Tukey’s multiple comparisons were used when group differences were significant. For each of the six NNS pairings (CN and TN), a McNemar’s test was used to determine whether respondents were more likely to correctly identify the substance as a NNS with TN as opposed to CN. A one-way repeated measures ANOVA was used to compare the mean number of correct identifications of NNS by TN and CN relative to the decoy NNS with multiple comparisons made using Tukey’s HSD. All differences were considered significant when *p* < 0.05. Strength of significant associations were examined using Spearman’s rho value.

## 3. Results

### 3.1. Respondent Demographics

The survey was delivered to 1243 students, with 493 respondent surveys being completed and used for data analysis. Participants who did not reply to the survey, did not open the survey, or completed less than 95% of the survey were not considered in the statistical analysis. A total of 453 respondents completed the survey in under 6 min (5.4 ± 3.4 min), with a small number (*n* = 40) taking longer to submit the completed online survey. Respondents who completed the survey consisted of 105 males and 388 females aged 18.9 ± 2.2 years old, with a BMI of 24.4 ± 4.8, and an ACT score of 23.7 ± 3.1 upon university admission. They consisted of Freshmen, Sophomores, Juniors, and Seniors representing 71.1, 17.9, 7.0, and 3.7% of respondents. Respondent nutrition training in prior classes was classified as either none, 1–3 credit hours, or 4–10 credit hours, representing 52.9, 40.2, and 7.0% of respondents. 

### 3.2. Ability to Write an NNS Definition

Respondent NNS definitions were examined and given one point each for the presence of words related to (1) chemical nature, (2) taste, (3) caloric content, and (4) non-nutritive quality, with a total score of four indicating the presence of all four components and zero indicating none of the components or that the respondent could not provide any definition. The overall NNS definition score was 1.1 ± 1.1. Written definitions that included none, one, two, three, or four of these NNS components were provided by 41.6%, 20.7%, 26.1%, 10.6% and 1.1% of respondents. As such, 62.3% of total respondents were unable to provide any definition or provided one or none of the four target components. NNS definition score was not associated with gender, age, BMI, ACT, completion of prior nutrition classes, or history of weight loss attempt. Freshman respondents had NNS definition scores (1.0 ± 0.1) that were significantly lower than sophomores (1.3 ± 0.1) or seniors (1.5 ± 0.3). Self-reported use of food labels was recorded by 55% of respondents whose NNS definition score was 1.2 ± 1.1 while non-label user NNS definition score was significantly lower (0.9 ± 1.0).

NNS definition score was correlated with the self-estimated frequency of NNS dietary intake (Table 1). However, data presented later in this study suggests that respondents had difficulty identifying the NNS they claimed to use in this survey. The poorest NNS definition scores were associated with those who did not know if they consumed them, and the best scores were observed for persons who claimed to never consume NNS (1.56 ± 1.04), although this group represented only 5.2% of respondents.

### 3.3. Ability to Name Examples of NNS from Memory with a Fill-in-the-Blank Format

After being provided with a working survey NNS definition for this study, respondents were asked to demonstrate their depth of NNS knowledge by writing the names of every NNS they knew from memory. A total of 576 potential words were provided, and respondents could name 1.0 ± 1.1 NNS from memory by TN or CN. Of respondents, 37.9% could name no NNS examples, 39.1% could name one NNS, 11.3% could name two NNS, 9.1% named three NNS, 1.5% named four NNS, and 0.4% named more than four NNS examples. With respect to CN, none, one, two, three and four of the NNS examples were identified by 44.0%, 37.9%, 10.3%, 5.9%, and 0.4% of respondents. With respect to TN, none, one, two, three, four, and seven of the NNS examples were identified by 38.1%, 39.7%, 10.3%, 9.9%, 1.0%, and 0.4% of respondents. The number of fill-in-the-blank TN examples (0.9 ± 0.9) was significantly greater than the number of CN examples (0.2 ± 0.6).

The influence of respondent demographics and habits on ability to name examples of NNS by TN and CN in the fill-in-the-blank exercise was also examined. NNS identification by TN was significantly better among females (0.9 ± 0.1) than males (0.6 ± 0.1). Ability to provide TN examples was also significantly influenced by having completed prior nutrition classes, with those having completed 0, 1–3, and 4 or more credits earning TN scores of 0.7 ± 0.8, 0.8 ± 0.9, and 1.2 ± 1.1, respectively. While the correlation between ACT and the number of NNS identified by TN was statistically significant, the Spearman’s correlation was 0.1151, therefore the association was very weak. TN association with BMI, age, and class was not statistically significant. CN identification was significantly associated with class rank. Seniors, Juniors, Sophomores and Freshman identified 1.0 ± 0.2, 0.9 ± 0.2, 0.7 ± 0.1 and 0.4 ± 0.1 NNS by CN, respectively. In contrast to TN, males were significantly better at identifying NNS by CN (0.8 ± 0.1) than females (0.5 ± 0.1). Ability to identify CN examples was also significantly associated with BMI, age, and ACT, however the Spearman’s correlation values were 0.1086, 0.1390, and 0.1365, respectively; therefore, these associations were very weak. Completion of prior nutrition class credit was not associated with a statistically significant ability to identify NNS by CN in the fill-in-the-blank exercise.

Respondents who reported use of food labels provided 1.2 ± 1.2 NNS examples from memory, while those not using food labels provided significantly fewer NNS examples (0.8 ± 0.9). Respondents who had tried to lose weight in the past and who had never tried to lose weight provided 0.9 ± 1.1 and 0.9 ± 0.9 NNS, which was significantly less than those who were currently trying to lose weight (1.2 ± 1.1 NNS). Ability to provide NNS examples from memory was best for those who attempted to never consume them (1.4 ± 0.8) and worst for respondents who did not know if they consume NNS (Table 2).

The number of each kind of specific NNS name written into the fill-in-the-blank portion of the survey was also examined. Responses classified as a TN included Splenda, Stevia, Sweet’N’ Low, Truvia, Equal, NutraSweet, and SweetOne, which were identified by 43.8%, 12.8%, 10.3%, 5.9%, 4.9%, 0.6%, and 0.2% of respondents, respectively. Responses classified as a CN included aspartame, sucralose, saccharine, erythritol, xylitol, acesulfame, cyclamate, and neotame; these were identified by 10.1%, 2.8%, 1.6%, 0.8%, 0.8%, 0.6%, 0.2%, and 0.2% of respondents. In the fill-in-the-blank portion of the survey, 6.1% respondents incorrectly identified “corn syrup” or “high fructose corn syrup” as being a NNS. 

### 3.4. NNS Recognition by TN, CN and Decoy Name Using a Click-Drag-Box Format

**Figure 2:**
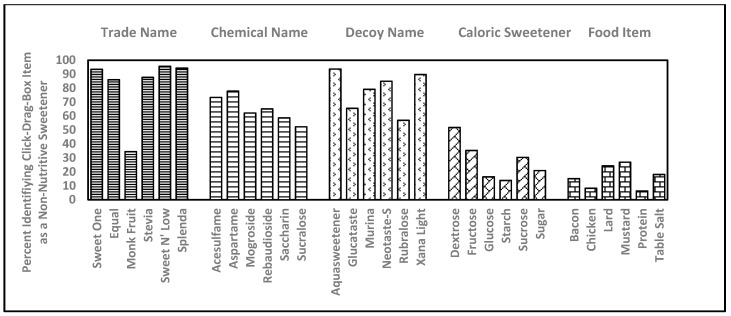
Percentage of respondents identifying 36 click-drag-box items within five categories as being examples of non-nutritive (artificial) sweeteners.

**Figure 3:**
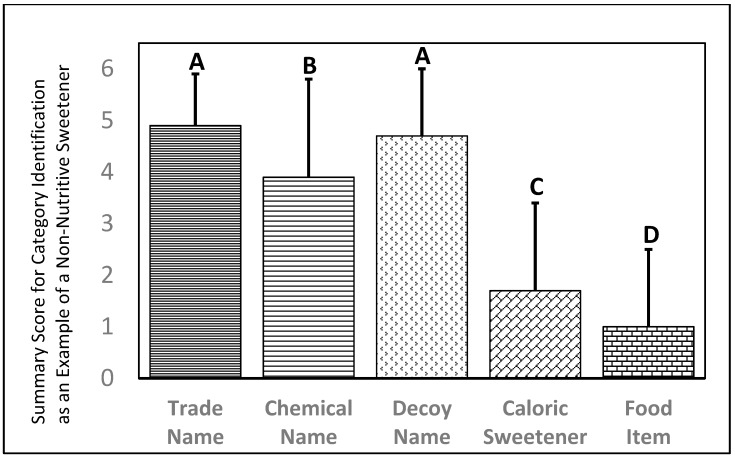
Respondent ability to sort items within different categories as being examples of non-nutritive (artificial) sweeteners (NNS) with a click-drag-box tool. Mean ± SD with statistical significance between groups indicated by differing column letters using Tukey’s HSD (*p* < 0.05).

Respondent ability to use a *click-drag-box* to successfully identify NNS (with familiarity prompting where they saw actual survey words) was used to evaluate relative ability to recognize six CN and their respective TN, a set of six decoy (false) NNS names, six common caloric sweeteners, and six food items (Figure 2). TN were correctly identified as NNS 4.9 ± 1.0 times while CN were correctly identified as NNS 3.9 ± 1.9 times (Figure 3). Decoy names were incorrectly identified as a being a true NNS 4.7 ± 1.3 times (Figure 3). The tendency to identify CN as a NNS was lower than the tendency to identify a decoy as being a true NNS. The tendency to incorrectly identify decoy names as being as true NNS was not significantly different from the tendency to correctly identify TN as being an example of a true NNS. Respondent ability to use a *click-drag-box* to identify NNS by TN ranged from a high of 95.7% for Sweet ‘N’ Low to 34.5% for Monk fruit (NNS sourced from a raw food were grouped as a TN in this study) with the average being 4.9 ± 1.0. Ability to use a *click-drag-box* to identify the NNS by CN ranged from a high of 73.3% for Aspartame to a low of 52.2% for Sucralose, with the average being 2.9 ± 2.2. McNemar’s test was used to show that respondents were significantly better able to identify *click-drag-box* NNS by TN relative to each paired CN for all six comparisons.

The decoy NNS names, Aquasweetener, Glucataste, Murina, Neotaste-S, Rubralose, and Xana Light were identified as a NNS by 93.7%, 65.5%, 79.1%, 84.6%, 56.9%, and 89.7% of respondents. The summary score for decoy names (4.7 ± 1.4) was not significantly different from TN, but was significantly greater than CN (Figure 3).

### 3.5. Recognition of Caloric Sweeteners and Food Items Using a Click-Drag-Box Exercise

As a reference, respondents were also asked to sort six caloric sweeteners and six food items (Figure 2). Caloric sweeteners were incorrectly identified as NNS with a summary score of 1.7 ± 1.7 (Figure 3). Specifically, dextrose, fructose, glucose, starch, sucrose and sugar, were improperly identified as being an NNS 51.2%, 35.3%, 16.4%, 13.8%, 30.4% and 21.0% of the time. Food items were incorrectly identified as NNS with a summary score of 1.0 ± 1.5. Specifically, bacon, chicken, lard, mustard, protein, and table salt were improperly identified as being an NNS 15.1%, 8.2%, 24.3%, 27.0%, 6.3%, and 18.1% of the time. The summary score for food items was significantly lower than for nutritive sweeteners, and both food items and caloric sweeteners were significantly less likely to be identified as an NNS than TN, CN or decoy NNS items.

## 4. Discussion

### 4.1. Clinical Implications of NNS Definition Knowledge

The average respondent in this study was able to identify 1.1 ± 1.1 of the four NNS characteristics used to evaluate the depth of their NNS definition. Among respondents, the best predictor of NNS definition depth was self-identified NNS avoidance by the respondents. With respect to NNS, respondents who “never consumed them” had a definition score of 1.6 ± 1.0 which was significantly greater than all other groups including those who “did not know” if they consumed them (0.9 ± 1.1). To avoid something, a respondent needs to know what it is they are avoiding, which presumably created a personal need for a base of information to facilitate NNS avoidance; these observations appear to reflect this. A similar establishment of dietary avoidance behaviors and a need for food content knowledge are associated with gluten and food allergies [17,18]. In the present study NNS definition score was not associated with BMI, gender, age, ACT, completion of prior nutrition classes, or history of weight loss attempts. Perhaps this reflects the younger age of the study population and their developing an understanding of weight management methods; in this regard, NNS understanding was better for Seniors than Freshman.

Analysis of NHANES two-day food and beverage recall information suggests that NNS intake was increased among those with completion of prior college degrees [19], however, this NHANES data did not determine whether participants knew if they consumed NNS. In contrast, the present study examined NNS intake knowledge among those in the process of completing a college degree in the sciences. Having taken prior nutrition courses could influence NNS knowledge and was significantly correlated with NNS definition score, but given that the best scores (1.5 ± 1.0 out of 4 NNS qualities) were only observed for students who had completed four or more nutrition course credits, one can say that most university students remained largely unable to provide a working NNS definition. Degree-holding RDNs, RNs, MDs, and other health professionals often need to communicate about NNS with clients; they presumably know more NNS examples than this undergraduate student population. However, as of July 2018, only 30.1% of the US population 25 years old and older had completed any sort of bachelor’s or higher degree [20], let alone a degree that promotes NNS training, use, or knowledge. The present study suggests that university students and the less educated general public are unable to confidently know what an NNS really is.

### 4.2. Clinical Implications of Using a Fill-in-the-Blank Exercise to Provide NNS Examples

In a marketing survey of 2133 respondents that examined factors related to building food brand trust, two items were described as extremely/very important: (1) “Transparent/doesn’t try to hide information” and (2) “Open about how food is made/what it contains” [21]. Respondents in the present study were significantly better at providing names of NNS by TN (0.8 ± 0.9) than by CN (0.2 ± 0.6), although 1/3 of respondents could not name a single NNS of any kind. As with NNS definitions, ability to name NNS examples was best among those who “never consumed them” implying a need to find and become familiar with examples of NNS and apply this information to an ingredient label (CN) or packaging (TN). 

This greater ability to name NNS by TN probably reflects the not-surprising success of marketing or advertising campaigns in the print, online, and televised media that promote market NNS name recognition. What ought to be surprising is the apparent naïveté which remains in this university student population, in spite of robust marketing attempts to improve TN NNS recognition ability and market loyalty to a specific TN. If one goal of labeling is to improve consumer knowledge of what people ingest, the present study suggests that perhaps ingredient labels should list NNS by both TN and CN if dietary transparency is a goal of ingredient labels and package labeling.

The NNS naïveté of respondents was also reflected by the fact that high fructose corn syrup was more likely to be given as an example of an NNS than five of the eight TN and seven of the eight CN examples provided in the fill-in-the-blank exercise. This observation presumably reflects respondent exposure to debate in the popular media regarding the suitability of high fructose corn syrup for human consumption. 

### 4.3. Clinical Implications of NNS Familiarity with a Click-Drag-Box Exercise

Respondents were far better at recognizing NNS by TN than by CN. NNS preferences change year-to-year based on market and consumer forces on a regular basis. The purpose of this investigation was not to try and determine which product is the “current” best or least recognized NNS, but primarily to look at the difference in ability to identify NNS by TN or CN. Given marketing and media exposure, it is not surprising that TN are better recognized. What is surprising is the lack of peer-reviewed literature that attempts to characterize this difference which could be important for interpreting trends in NNS consumption and their implications for nutritional health. 

The degree to which the name of a TN or CN is suggestive of its NNS nature could be an important factor that could skew perceived consumer ability to identify NNS on an ingredient list or food/beverage package labeling. Six of the randomly ordered click-drag-box items were decoy NNS names that evaluated suggestiveness in terms of tendency that a word might be chosen to be an NNS simply because it sounded like it could be a NNS. Decoy names were frequently identified by respondents as being real NNS (Figure 2), suggesting that a “name” can potentially represent a large part of what could be falsely interpreted by clinicians, researchers, and the general public as NNS “knowledge”. Respondents were significantly more likely to identify a decoy name as being a NNS than the CN, which would appear on the ingredient list. Future investigations of consumer knowledge of NNS names may want to consider this tendency when exploring NNS usage and familiarity in the general public.

Caloric sweetener items were included in the click-drag-box to provide a reference to basic nutrition knowledge. Dextrose was the food item most likely to have been identified as an NNS (51.7%). Respondents were apparently making an effort to provide sincere survey answers, as the CN ‘sucralose’ was properly identified as an NNS by 52.2% of the time and the close but different caloric sweetener ‘sucrose’ was improperly identified as an NNS 30.4% of the time. 

Food items were also included in the click-drag-box to provide a reference to basic nutrition knowledge under the assumption that these items would not be frequently chosen to represent NNS. In this regard food items (1.7 ± 1.7) were significantly less likely to be chosen to represent an NNS than TN, CN, decoy items or caloric sweeteners. This suggests that respondents were generally knowledgeable about basic nutrition and made a sincere attempt to sort the items properly into their respective the click-drag-box (NNS or Not an NNS). The observation that some food items were chosen to reflect NNS could represent a failure to understand survey instructions, survey completion insincerity, or respondent haste to complete the click-drag-box portion of the survey. However, the survey design and IRB Human Subjects Committee approval did not permit the investigators to interview respondents post-hoc to determine the answer to this question.

### 4.4. Study Limitations 

Respondents who knew nothing about NNS or nutrition may have chosen to ignore the survey invitation or may have only partially completed the survey, in which case their data was not considered in the statistical analysis. If this were true, less knowledgeable or less confident respondents would not have participated in this survey and the present study would have measured NNS understanding mostly in those with a greater depth of NNS knowledge. If this were true, the typical American university student would be even less educated about NNS than the respondents in the present survey. 

It is possible that respondents could have used online information, friends, or book sources to improve their ability to complete this survey and provide an apparent improved NNS knowledge. However, several of the NNS items (monk fruit, mogroside, and rebaudioside) were present in the randomly populated click-drag-box exercise, but not provided in any of the fill-in-the-blank exercise responses, which suggests (but does not prove) that respondents did not look things up externally to provide better answers to this survey. In the present study, 91.9% of respondents completed the survey in one single attempt, requiring 5.6 ± 3.4 min, which would give little time for respondents to consult other sources and skew their perceived NNS knowledge score making apparent NNS knowledge better than described here. Furthermore, the completion time for the present study was similar to the 5.8 ± 3.0 min duration required to complete a 2013 pilot trial of NNS knowledge after 22 of the 720 respondents requiring over 30 min were considered separately [16]. Therefore, the present study is presumed to represent a sincere reflection of respondent NNS knowledge without the use of other sources to complete this online survey.

The observations of the present study are assumed to reflect national trends. This survey of NNS knowledge was completed at a teaching-focused Midwestern university with a total enrollment of 8700 students, with two-thirds of students originating from Minneapolis-Saint Paul, Rochester, Milwaukee, or Chicago metropolitan areas. For reference, the American College Health Association Report for Fall 2018 indicates that 58% of students had a BMI of 18.5–24.9 and that 36.0% of US university students are currently attempting to lose weight [13]. These values are similar to those observed in the present study, which is presumed to be reflective of typical university populations.

## 5. Conclusions

This study was limited to university students seeking a career in the sciences and healthcare and suggests that respondents were unable to provide an effective NNS definition or examples, had a limited ability to identify NNS by TN, and an even lower ability to identify NNS by CN. Since TN and decoy NNS names were identified at similar percentages, true NNS depth of knowledge was surprisingly poor in this regard. This study assumed that respondents would have a greater knowledge of NNS than the general population, because nutrition knowledge could be a part of their future career. Future studies may wish to determine if older Americans could have a different level of NNS understanding.

As a practical clinical application, this study suggests that information found on an ingredient label is not helpful for consumer understanding of what they consume. Understanding what you ingest is different from what is reported in NHANES data sets and UPC estimates of national consumption because it reflects consumer knowledge about a topic that has received a great deal of attention in the peer-reviewed literature and media. The appearance of TN on beverage/food packaging is central to the marketing of NNS and the focus of NNS product marketing education programs that seek to develop brand-name loyalty among consumers. Given that NNS contents on the ingredients label need only be identified by CN or raw food item (i.e., monk fruit), this study suggests that people have little of the working knowledge needed to understand if they consume NNS in their diet. This observation could be important for interpreting the effect of beverage/food label changes in the United States and the effects that dietary NNS have on human health. The question of whether consumers are aware of what a NNS “is” or whether they “consume” NNS should be part of studies that evaluate potential NNS health benefits or relative NNS safety as part of the diet.

## Figures and Tables

**Table 1:** Respondents who self-reported NNS avoidance provided NNS definitions with greater depth of knowledge.

Self-Described NNS Use:	Percent	Definition Score
I do not know if I consume them	18.9	0.9 ± 1.1 ^A^
I never consume them	5.2	1.6 ± 1.0 ^B^
Once or more each day	21.1	1.0 ± 1.1 ^C^
Once or more each week	37.8	1.1 ± 1.1 ^B,C^
Once or more each month	10.2	1.4 ± 1.2 ^A,B^
Less than once each month	6.3	1.1 ± 1.1 ^A,B,C^

Statistical significance (*p* < 0.05) between categories is indicated by differing superscript letters.

**Table 2:** Respondents who self-reported avoidance of NNS in the diet provided a greater number of NNS examples from memory in a fill-in-the-blank format.

Self-Described NNS Use:	Percent (%)	Number of NNS Examples
I do not know if I consume them	18.9	0.8 ± 0.9 ^D^
I never consume them	5.2	1.4 ± 0.8 ^A,B^
Once or more each day	21.1	0.9 ± 1.0 ^C,D^
Once or more each week	37.8	1.0 ± 1.1 ^B,C^
Once or more each month	10.2	1.4 ± 1.2 ^A^
Less than once each month	6.3	1.2 ± 1.1 ^A,B,C^

Statistical significance (*p* < 0.05) between categories is indicated by differing superscript letters.
